# The Hypotensive Role of Acupuncture in Hypertension: Clinical Study and Mechanistic Study

**DOI:** 10.3389/fnagi.2020.00138

**Published:** 2020-05-25

**Authors:** Hao Fan, Jing-Wen Yang, Li-Qiong Wang, Jin Huang, Lu-Lu Lin, Yu Wang, Na Zhang, Cun-Zhi Liu

**Affiliations:** ^1^Acupuncture Research Center, School of Acupuncture-Moxibustion and Tuina, Beijing University of Chinese Medicine, Beijing, China; ^2^Department of Acupuncture and Moxibustion, Beijing Hospital of Traditional Chinese Medicine Affiliated to Capital Medical University, Beijing, China; ^3^School of Acupuncture-Moxibustion and Tuina, Shandong University of Traditional Chinese Medicine, Jinan, China

**Keywords:** acupuncture, blood pressure, clinical study, hypertension, mechanism study

## Abstract

As a component of traditional Chinese medicine (TCM), acupuncture has the potential to lower blood pressure (BP) in patients with hypertension. Emerging evidence indicates that the acupuncture-induced inhibition of high BP occurs through the activation of the pathway in the afferent, central, and efferent pathways. An increasing number of studies have demonstrated that acupuncture not only activates distinct brain regions under conditions of hypertension caused by an imbalance between the sympathetic and parasympathetic systems but also modulates neurotransmitters in related brain regions to alleviate the autonomic response. The activity of these pathways can be assessed by injecting agonists or inhibitors or by performing neurotomy. This review focuses on the clinical and mechanistic studies of acupuncture in modulating BP, which might provide a neurobiological foundation for the effects of acupuncture. Although many mechanisms underlying the effects of acupuncture on cardiovascular function have been identified, further investigation is warranted.

## Introduction

Hypertension has an overall prevalence of 46% in the general adult population in the United States and is thus the dominant preventable risk factor for premature death and disability worldwide (Table 1, Forouzanfar et al., 2015; Muntner et al., 2018). Hypertension causes nearly 9.4 million deaths per year from cardiovascular-related mortality (Yang et al., 2017) people aged ≥60 years constituted >16.2% of the Chinese population in 2017, but the percentage predicted by the UN will increase to 35.1% by the year 2050 (United Nations, 2017). Currently, there are numerous recommendations for controlling hypertension, including improving lifestyle, such as doing more exercise, keeping a balanced diet, and having pharmacological treatment. However, drugs that nonspecifically block receptors may have multiple side effects, and most individuals find it challenging to change their long-formed lifestyle; therefore, there is a growing need to explore alternative medical procedures for treating hypertension worldwide (Li and Longhurst, 2010; Li et al., 2010a).

**Table 1:** Percentage of American adults meeting the definition for hypertension and recommended antihypertensive medication.

	2017 ACC/AHA Guideline		JNC7 Guideline	
	Hypertension	Recommended Anti-hypertensive Medication	Hypertension	Recommended Anti-hypertensive Medication
Overall	45.6 (43.6–47.6)	36.2 (34.2–38.2)	31.9 (30.1–33.7)	34.3 (32.5–36.2)
Age group (years)				
20–44	24.0 (21.8–26.2)	12.5 (11.2–13.9)	10.5 (9.4–11.7)	12.2 (10.9–13.6)
45–54	47.1 (44.4–49.8)	33.4 (30.8–36.1)	29.5 (27.0–32.2)	32.7 (30.1–35.4)
55–64	66.6 (63.6–69.5)	58.2 (54.9–61.4)	52.4 (49.1–55.7)	55.0 (62.0–58.0)
65–74	75.6 (73.4–77.6)	74.1 (71.4–76.6)	63.6 (60.2–66.9)	66.9 (63.7–69.9)
≧75	82.3 (79.2–85.0)	82.3 (79.2–85.0)	75.1 (71.9–78.1)	78.5 (74.7–81.8)
Men	48.6 (45.9–51.3)	37.3 (34.9–39.8)	32.0 (29.8–34.3)	34.8 (32.4–37.3)
Women	42.9 (40.7–45.1)	35.1 (33.1–37.3)	31.8 (29.8–33.8)	33.8 (31.8–35.9)
History of cardiovascular disease	79.3 (75.6–82.6)	79.3 (75.6–82.6)	72.1 68.8–75.3)	75.7 (72.7–78.4)

*Values are % of American adults (95% confidence interval). ACC/AHA, American College of Cardiology/American Heart Association; JNC7, Seventh Report of the Joint National Committee on Prevention, Detection, Evaluation and Treatment of High Blood Pressure*.

Acupuncture has been reported to have roles in treating hypertension (Abdi et al., 2017). This ancient treatment technique in traditional chinese medicine (TCM) has a much lower (0.13%) incidence of side effects (MacPherson et al., 2001) than traditional Western medicine (WM). In recent years, several randomized controlled trials have shown that acupuncture alleviates blood pressure (BP) in patients (Wang et al., 2016; Zheng et al., 2016). Animal experiments (Yang et al., 2017) have also shown that acupuncture has a significant effect on the BP of spontaneously hypertensive rats (SHRs). Since Besedovsky and Sorkin (1977) proposed the neuroendocrine network theory, scholars have examined this research topic, which provides new clues for understanding hypertension; the complex network of neurotransmitters and the nervous system are often disordered in the context of hypertension. Experts have shown that acupuncture plays a role in lowering BP through effects on the nervous system [including the afferent pathway, central nervous system (CNS), and efferent pathways] and neurotransmitters. Hence, this review summarizes the results of clinical and mechanistic studies to explain the antihypertensive effect of acupuncture.

## Clinical Studies on Acupuncture for Hypertension

### Antihypertensive Effect of Acupuncture

Most studies have shown that acupuncture can decrease BP in hypertensive patients. Zheng et al. (2019) reported that compared with participants who received sham acupuncture and those on a waiting-list group, individuals who received active acupuncture (three sessions per week, 6-week treatment) showed better improvements in systolic blood pressure (SBP) and diastolic blood pressure (DBP) at weeks 6, 9 and 12. These data suggest that although the beneficial effects do not appear immediately, they persist for a longer period. Also, a meta-analysis (Chen et al., 2018) demonstrated that acupuncture used together with antihypertensive drugs achieve better results than antihypertensive drugs alone in reducing SBP and DBP. Similar results were observed in experiments that compared a combination of acupuncture and medication to that of sham acupuncture and medication in the reduction of SBP and DBP. These findings are supported by systematic reviews (SRs). Two SRs (Zhang et al., 2012; Zhao et al., 2015) found that acupuncture combined with WM had better outcomes than WM alone in reducing SBP. One SR (Zhao et al., 2015) found that a combination of acupuncture and WM was superior to WM alone in treating SBP and DBP, concerning efficacy rate. These data indicate that acupuncture is effective at reducing BP in patients with hypertension.

However, trials estimating the BP-lowering effects of acupuncture have shown diverse results. Although the Stop Hypertension With the Acupuncture Research Program (SHARP) was conducted by the United States, this acupunctural trial’s real effect of antihypertension is still debatable (Macklin et al., 2006). There was no significant difference in BP reduction, which between the acupuncture group and the sham acupuncture group from its baseline to 10 weeks. The reason why no such difference may be the frequency (the number of acupuncture treatments per week). Although these experimental results are controversial, however, most of the other experiments indicate that acupuncture plays a role in reducing BP.

### Acupoint-Specific Effect of Acupuncture on Hypertension

**Figure 1:**
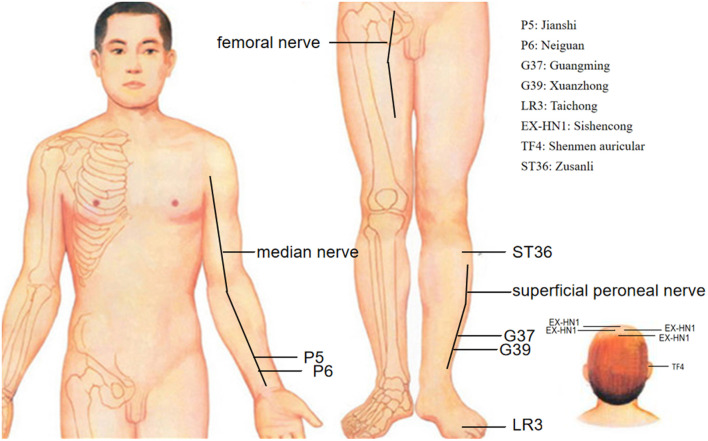
Effective and ineffective acupoints in acupuncture inhibition of hypertension. • Effective acupoints; ° Ineffective acupoints.

As acupuncture has garnered worldwide acceptance, it is important to define and study acupoints, which are located on a conceptual model of meridians and collaterals. This concept indicates that meridians make the human body an organic whole, and connect the viscera, body surface, and body parts (Bianco, 2019). Acupoints are the site through which the Qi of the internal organs and channels is transported to the body surface. These acupoints are used to transmit feedback, induce stimulation, and regulate physiologic functions. Chinese medicine (CM) identifies 12 bilateral meridians and eight extra meridians that connect internal splanchnic organs to external regions (Cheng et al., 2015). Hypertensive patients without medication received electroacupuncture (EA) at P5-6+ST36-37 (Neiguan-Jianshi+Zusanli-Shangjuxu, Figure 1) and then were assessed with 24 h by ambulatory BP monitoring. After treatment for 8 weeks, these patients showed significant reductions in SBP/24 h and DBP/24 h. Patients treated at the control acupoints G37-39+Li6-7 (Guangming-Xuanzhong+ Pianli-Wenliu, Figure 1) showed no reductions in SBP/24 h or DBP/24 h (Li et al., 2015). Similarly, while exercising, healthy individuals had inhibited stress-induced increases in SBP, mean blood pressure (MBP) and the double product (SBP×heart rate, reflecting myocardial oxygen consumption) following 30 min of EA at the P5–6 acupoints (Figure 1; Longhurst et al., 1980; Li et al., 2004). However, EA at the G37–39 (Figure 1) control acupoints did not alter the exercise-related increase in BP or the double product. The strong effect at P5–6 on BP and the absence of an influence of acupuncture at G37–39 provide further evidence for the acupoint-specific effects of acupuncture on hypertension, suggesting a close relation between acupoint specificity and meridians.

## Mechanistic Studies on Acupuncture for Hypertension

In recent years, mechanistic studies have greatly improved our understanding of hypertension. Our previous study (Wang et al., 2018) showed that acupuncture exerts an antihypertensive effect by ameliorating oxidative stress and the redox-sensitive pathway in the rostral ventral lateral medulla (RVLM) of SHRs. At the same time, there is increasing mechanistic research on acupuncture therapy for hypertension. The effects of acupuncture on hypertension encompass a holistic regulatory process. Anatomically, the afferent pathway, efferent pathway, CNS, and neurotransmitters can be distinguished. Hence, we need to explain why acupuncture lowers BP from the perspective of each of these components.

### Afferent Pathway

#### The Beginning of the Afferent Pathway

Acupuncture stimulates specific but poorly defined sites called acupoints (Kim et al., 2017), and this is the starting point for research on the mechanism of acupuncture. TCM describes the special communication between each acupoint and a specific visceral organ; an acupoint can reflect the status of specific visceral organs, and visceral disorders can be treated by manipulating these acupoints (Stux and Pomeranz, 1998; Rong et al., 2011). Acupoints are a part of the afferent pathway. Although there has been a great deal of effort in identifying acupoints, their anatomical structures remain unknown. Acupoint specificity was regarded as a major scientific issue in acupuncture practice at the Society for Acupuncture Research international symposium in 2007 and the American Association of Acupuncture conference in 2010.

**Figure 2:**
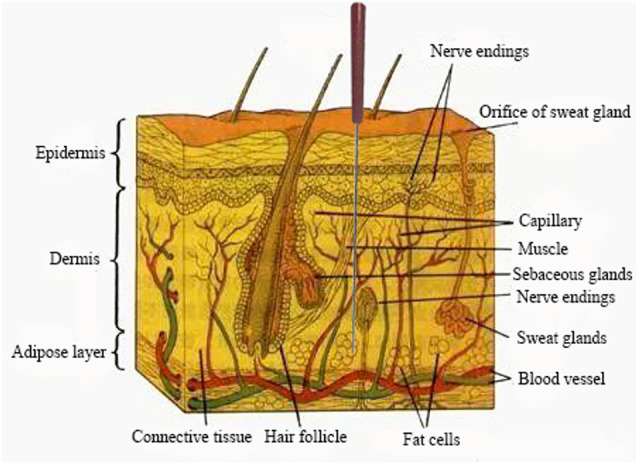
A representative region that is abundant in nerve endings, blood vessels etc. In response to acupuncture stimulation.

Acupoints are always located in areas abundant in nerves, blood vessels, and lymph vessels, and under physiological conditions, these sites contain densely distributed nerve endings and receptors, mast cells and blood vessels and highly concentrated neural and neuroactive components (Figure 2; Zhang et al., 2012). It is generally accepted based on circumstantial evidence that acupoints become hypersensitive when pathological conditions develop in visceral organs (Ben et al., 2012) and have higher electrical conductance than the surrounding tissue (Ahn and Martinsen, 2007; Ben et al., 2012). Similarly, a previous study in a rat model of hypertension reported that the high conductance at acupoints is attributed to the release of the neuropeptide substance P (SP) and calcitonin gene-related peptide (CGRP; Fan et al., 2018). This study would help solve some of the controversial issues concerning the electrical properties of acupoints. Kim et al. (2017) showed that consistent with the physiological characteristics of acupoints, neurogenic spots caused by activation of somatic afferents during visceral disorders: (1) are most frequently located in the same anatomical location as traditional acupoints; (2) have high electrical conductance; and (3) show mechanical hypersensitivity.

#### Afferent Fibers

It is generally acknowledged that stimulation of the skin and muscles through acupuncture either electrically or with noxious or non-noxious stimuli can induce various somatomotor and autonomic responses (Kagitani et al., 2010). This implies that acupuncture acts by exciting cutaneous and/or muscular afferent nerve fibers. Studies show that manual acupuncture-like stimulation of a hindleg in rats modifies BP, heart rate, and the secretion rate of adrenal medullary catecholamine hormones (adrenaline and noradrenaline; Uchida et al., 2008). Neuronal activity underlying the acupoints is eliminated by surgical denervation of the median and ulnar nerves or inactivating TRPV receptors on small diameter afferent fibers of the median and ulnar nerves with resiniferatoxin (Fan et al., 2018). The effects of acupuncture were lost after the somatic afferent nerves [femoral nerve (Figure 1) and sciatic nerve] innervating the needle insertion site were severed surgically. Our research data showed (Wang et al., 2018) that surgical or chemical ablation (capsaicin) of sciatic nerve signals abolishes the antihypertensive effect of acupuncture at LR3 (Taichong, Figure 1). These findings reveal that afferent fibers are involved in the ability of acupuncture to lower BP. Afferent fibers are damaged, such as trauma, which may influence the hypotensive effect of acupuncture.

Previous studies have shown that somatosensory afferents are stimulated during acupuncture treatment. There are different types of afferent fibers, including thick myelinated Aα and Aβ (groups I and II), thin myelinated Aδ (group III), and thinner unmyelinated C (group IV) fibers. According to the study by Tjen-A-Looi (Tjen-A-Looi et al., 2005), unmyelinated fibers in the median nerve activated by P5–6 acupoint stimulation also participate in the inhibitory effects of EA on cardiovascular responses. Investigators (Uchida et al., 2003) have concluded that group III fibers are involved in the actions of EA. The inhibitory effect of acupuncture at P5–6 (Figure 1) on cardiovascular excitatory reflexes involves both thinly myelinated and unmyelinated fibers (Tjen-A-Looi et al., 2004).

### Efferent Pathway

The efferent pathway mainly depends on the sympathetic and vagus nerves, which originate in the spinal cord or a certain nucleus of the brain and form the peripheral nervous system of vertebrates. The sympathetic and vagus nerves, as the operating system of humans, permeate all organ systems, and are thus involved in virtually all diseases (Ziemssen and Siepmann, 2019). Acupuncture influences BP mainly through these two types of efferent nerves.

The sympathetic mechanism of action modulated by acupuncture was shown through *in vivo* experiments (Li et al., 2002; Tjen-A-Looi et al., 2003, 2004): (1) EA at P5–6 along the pericardial meridian reduces sympathoexcitatory-related increases in BP; and (2) EA induces the inhibition of evoked premotor sympathetic RVLM neuronal discharge. These data suggest that acupuncture lowers BP through sympathetic nerves.

Clinical trials and animal experiments have been conducted to investigate cardiovascular vagal regulation by acupuncture (Hsu et al., 2007; La Marca et al., 2010; Gao et al., 2011, 2012). Auricular EA stimulation was found to have a positive effect on respiratory sinus arrhythmia adjusted for tidal volume, indicating an increase in vagal activity, and therefore, this modality has potential as a treatment or preventive method for diseases such as hypertension and arrhythmia (La Marca et al., 2010). Hsu Hsu et al.’s (2007) study, acupuncture at TF4 (shenmen acupoint, Figure 1) and EX-HN1 (Sishencong acupoint, Figure 1) reduced BP, slowed the heart rate and activated the vagal nerves. Acupuncture at the auricular point “Heart” in anesthetized Sprague–Dawley rats had a significant inhibitory effect on arterial pressure and heart rate by activating the vagus nerve (Gao et al., 2011); therefore, acupuncture led to more vagal and less sympathetic activity, which contributed to the reduction of hypertension. Activated sympathetic and decreased vagal activities are an attractive target for future therapies for hypertension.

### Central Nervous System

**Figure 3:**
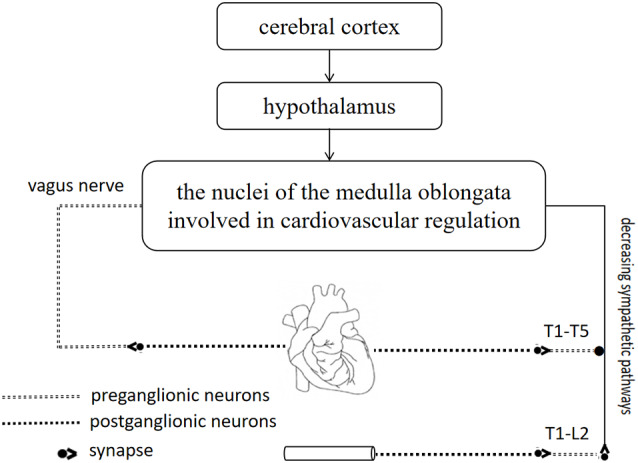
The importance of central nervous system (CNS) in cardiovascular regulation. The cerebral cortex and hypothalamus are closely related to the nuclei of the medulla oblongata, where is coordinated and integrated with input from baroreceptors and chemoreceptors. Sympathetic neurons and vagus nerve are involved in cardiovascular regulation.

The importance of the CNS in cardiovascular regulation is well established (Figure 3; Guyenet, 2006; Malpas, 2010). The CNS is mainly composed of the spinal cord and the brain, which are recognized as the most important and complicated parts of the nervous system (Wang et al., 2018). Acupuncture is effective at improving BP because it affects the CNS (Hua and Jie, 2019).

#### Spinal Cord

The spinal cord has various functions, including conveying afferent sensory information received from the peripheral nervous system to the brain (England, 2008; Joshua, 2019), transmitting signals from the motor cortex of the brain to the periphery (Kinaan, 2019) and serving as the center of reflex responses (Chu and Ng, 2018). Reflex responses that lead to activation of somatic and visceral afferents are integrated into cardiovascular supraspinal regions and the spinal cord. Outputs are relayed by the spinal cord from the CNS to effector organs involved in cardiovascular reflex regulation (Longhurst, 2003). One main component of the spinal cord associated with hypertension is the sympathetic nerve pathway.

Spinal sympathetic preganglionic neurons (SPNs) are mainly located within the lateral horns of spinal gray matter in spinal segments T1-L2. SPN axons exit the spinal cord through ventral roots and synapses on the sympathetic ganglionic neurons within the paravertebral chain ganglia or prevertebral ganglia (Krassioukov and Weaver, 1995). Furthermore, opioid and nociceptin-like immunoreactivities exist in the spinal sympathetic region (Dun et al., 1997; Pomeranz and Cheng, 1979). EA reduces visceral sympathoexcitation *via* opioid and nonopioid (nociceptin) mechanisms in the spinal cord dorsal horn and intermediolateral column (IML). Thus, this EA action in the IML shows that acupuncture also modulates sympathetic outflow from the spinal cord (Tjen-A-Looi et al., 2004; Zhou et al., 2009), suggesting that sympathetic nerves in the spinal cord play a role in BP regulation.

There are few reports on sympathetic nerve blockage of the spinal cord in hypertension, and such investigations have mainly focused on spinal cord injury (SCI) and drug inhibition. In a rat model of upper thoracic SCI has been established, rats with severe hypotension and bradycardia exhibited classic characteristics of a sympathetic nerve block (Krassioukov and Claydon, 2006; Krassioukov et al., 2007). In terms of drug inhibition of the sympathetic ganglion, NaCl was administered *via* intracerebroventricular infusions of 0.6 mol/l or 1 mol/l after sympathetic ganglionic blockade with chlorisondamine to assess the contribution of sympathetic nerve activity (SNA) to NaCl-induced pressor responses (Blaustein et al., 2012; Stocker et al., 2013). Stocker et al. (2015) showed that the intravenous injection of chlorisondamine decreased the mean arterial pressure (MAP), SNA, tachycardia, and pressor response induced by 0.6 mol/l and 1.0 mol/l NaCl. In another study (Ma et al., 2004), renal SNA and arterial pressure were recorded in anesthetized R^+^A^+^ mice (transgenic mice overexpressing renin and angiotensinogen) and littermate control mice before and after sympathetic ganglionic blockade with chlorisondamine. Ganglionic blockade reduced the SNA and BP in R^+^A^+^ mice. A literature review (Houghtling and Bayer, 2002; Li et al., 2013; Na et al., 2014; Lowrance et al., 2016) revealed that, in addition to surgical removal of superficial cervical sympathetic ganglia, the other sympathetic ganglia can be treated with intravenous or intraperitoneal injections of antagonists. The above experiments showed that blocking the sympathetic nerves in the spinal cord leads to a drop in BP. However, this method of blocking sympathetic ganglia in the spinal cord has the disadvantage of systemic rather than specific inhibition. The spinal cord has been shown to play a role in sympathoexcitatory responses including reflex responses and sustained elevated BPs that are reduced with acupuncture (Zhou et al., 2009). The above studies provide a theoretical basis for the mechanism of acupuncture.

#### Brain

The brain plays a very important role in the maintenance of BP homeostasis in the CNS (Drummond, 2019). The brain can adjust various components of the cardiovascular system to maintain homeostasis by modulating the release of critical hormonal factors and neurotransmitters, and the connections between brain nuclei (Rahmouni, 2016). Numerous types of cardiovascular nucleus dysfunction can affect BP. Here, we provide a review of the existing literature supporting a potentially important role for different brain nuclei in BP dysfunction, including the arcuate nucleus (ARC), midbrain ventrolateral periaqueductal gray (vlPAG), and RVLM.

**Figure 4:**
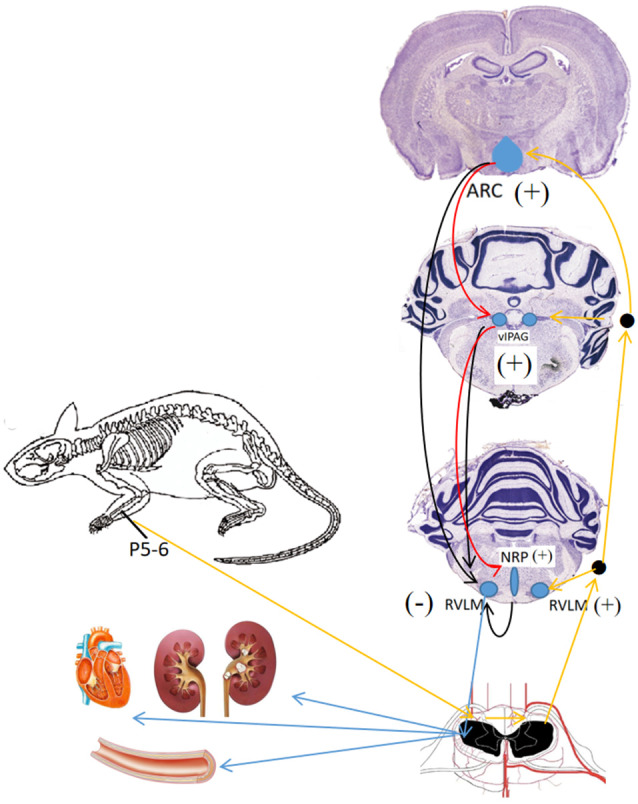
Neuronal pathways and mechanisms underlying the effect of acupuncture for different brain nuclei in blood pressure (BP) regulation. Black arrows, →, indicate inhibition effect from ARC, vIPAG, NRP; red arrows, 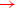, indicate activation effect from ARC, blue arrows, 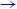 indicate efferent projections from different brain nuclei to heart, blood vessels and kidney; orange arrows, 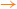, indicate afferent input from P 5–6 acupoints.

The ARC, located at the bottom of the hypothalamus, surrounds the ventral part of the third ventricle extending from the retrochiasmatic to the premammillary regions (Chronwall, 1985). The first direct evidence implicating the ARC in BP control derives from the work of Brody et al. (1984), which showed that direct electrical stimulation of the ARC evokes a frequency-dependent increase in arterial pressure and renal, mesenteric, and vascular resistance in anesthetized and conscious rats. Studies by Li et al. (2009) have shown that EA at P5–6 (Figure 1) activates ARC neurons, which then excite the vlPAG and subsequently inhibit cardiovascular sympathoexcitatory neurons in the RVLM. Micropipettes were inserted into the ARC, vlPAG, and RVLM for neural recording to determine whether or not the ARC inhibits RVLM activity directly or indirectly. The caudal vlPAG was shown to be required for the ARC-mediated inhibition of RVLM neuronal activation that is subsequently inhibited by EA. Although there are direct projections from the vlPAG to the RVLM, it remains unclear whether an indirect pathway through the nucleus raphe pallidus (NRP) plays an important role in vlPAG-RVLM cardiovascular modulation. EA at P5–6 for 30 min increased in the NRP response to splanchnic nerve (SN) stimulation, an effect that could be blocked by the microinjection of kynurenic acid (KA, a glutamatergic antagonist) into the caudal vlPAG. Also, the reflex increase in BP induced by bradykinin application to the gallbladder and the RVLM cardiovascular parasympathetic neuronal response to SN stimulation was inhibited by the injection of DL-homocysteic acid (DLH, a glutamate agonist) into the vlPAG, and these responses were reversed by injecting KA into the NRP. The results show that EA activates the vlPAG, which in turn excites the NRP to then inhibit RVLM parasympathetic neurons and reflex cardiovascular sympathoexcitatory responses (Figure 4; Li et al., 2010b).

### Neurotransmitters Responsible for Acupuncture-Mediated BP Regulation

Neurotransmitters are involved in the action of acupuncture on modulating BP (Flachskampf et al., 2007). Similarly, a series of studies have shown that several neurotransmitters participate in the inhibitory effects of acupuncture (Tjen-A-Looi et al., 2007, 2009; Guo et al., 2008; Fu and Longhurst, 2009).

Recent research and available evidence show that acupuncture activates distinct brain regions in cases of diseases caused by imbalances between sympathetic and parasympathetic activity and modulates neurotransmitters in related brain regions to alleviate autonomic responses (Flachskampf et al., 2007; Li Q. Q. et al., 2013). EA inhibition of reflex autonomic responses in cats is related to the activation of μ- and δ-opioid receptors in the RVLM, suggesting that endorphins, enkephalins and perhaps endomorphins, except for dynorphin, are responsible for the EA-mediated modulation of cardiovascular responses (Li et al., 2001). Improving the effects of EA at ST36 (Zusanli) on gastric motility might activate central opioids that, in turn, inhibit sympathetic outflow (Yin et al., 2010). Although acupuncture plays a significant role in decreasing BP and heart rate in pentobarbital-anesthetized rats, these responses are related to the activation of GABAergic neurons instead of opioid neurons (Uchida et al., 2008). Uchida et al. (2010) noted that opioid receptor-mediated transmission can be unrelated to the antihypertensive effect of acupuncture-like stimulation. The findings suggest that acupuncture may be related to different neurotransmitters, which is following the holistic view of acupuncture in TCM theory.

EA activates enkephalinergic neurons and endorphinergic neurons in several brain areas that regulate BP, including the ARC, vlPAG, and RVLM (Guo and Longhurst, 2007; Li et al., 2010). By this fact, *Longhurst* (Guo et al., 2004; Guo and Longhurst, 2007; Guo et al., 2008) found enkephalinergic neurons in the RVLM and endorphinergic neurons in the ARC that project directly to the RVLM; both neurotransmitter systems are activated by EA. Moreover, a previous study (Guo and Longhurst, 2010) showed the presence of reciprocal glutamatergic projections (excitatory neurotransmitters) between the ARC and vlPAG that participate in inhibiting elevations in BP. Additionally, data suggest that these reciprocal projections may include a cholinergic component in the ARC but not necessarily in the vlPAG. These findings also illustrate that connections in different brain regions are mediated by different neurotransmitters involved in regulating BP, including both excitatory and inhibitory neurotransmitters.

The sympathetic nervous system, *via* norepinephrine, regulates adrenergic receptor (AR) expression, and sympathetic activation causes sustained increases in BP by enhanced norepinephrine release. The long-lasting inhibition of sympathetic activity by EA was confirmed in EA-treated hypertensive patients with decreased levels of norepinephrine and renin (Cheng et al., 2015). Our previous study (Yang et al., 2017) indicated that renal sympathetic activation-induced upregulation of epinephrine, norepinephrine, and renin content, which were attenuated by acupuncture in SHRs. The decreased norepinephrine we observed might be involved in the beneficial effect of acupuncture on hypertension.

## Conclusion

An increasing amount of evidence shows that the effect of acupuncture on reducing BP occurs through various pathways, including acupoints, afferent nerves, the CNS, efferent nerves, and neurotransmitters. However, it remains unclear whether different pathways are activated by specific acupoints from the same meridian, including local points and distant points, or by acupoints from different meridians. Rigorous clinical studies and mechanistic studies are required to design future protocols for using acupuncture to lower BP. Such research would allow us to understand the relationship between acupoints and BP. Thus, acupuncture, as a general alternative therapy, can be used appropriately to treat hypertension.

## Author Contributions

HF, JH, and C-ZL were responsible for the study concept and design. L-QW and YW participated in collecting literature. HF and L-LL wrote the draft manuscript. J-WY and NZ were responsible for revising the manuscript.

## Conflict of Interest

The authors declare that the research was conducted in the absence of any commercial or financial relationships that could be construed as a potential conflict of interest.

## Abbreviations

ARC: arcuate nucleus
BP: blood pressure
CGRP: calcitonin gene-related peptide
CM: Chinese medicine
CNS: central nervous system
CVD: cardiovascular disease
DBP: diastolic blood pressure
DLH: DL-homocysteic acid
EA: electroacupuncture
EX-HN1: sishencong acupoint
IML: intermediolateral column
KYN: kynurenic acid
LR3: Taichong acupoint
MAP: mean arterial pressure
MBP: mean blood pressure
NRP: nucleus raphe pallidus
P5: Jianshi acupoint
P6: Neiguan acupoint
RVLM: rostral ventral lateral medulla
SBP: systolic blood pressure
SCI: spinal cord injury
SHR: spontaneously hypertensive rat
SN: splanchnic nerve
SNA: sympathetic nerve activity
SP: substance P
SPN: sympathetic preganglionic neuron
SR: systematic review
TCM: Traditional Chinese Medicine
TF4: shenmen acupoint
vlPAG: ventrolateral periaqueductal gray
WM: Western medicine.
